# Iterative Learning Control Without Resetting Conditions of an Algorithm Based on a Finite-Time Zeroing Neural Network

**DOI:** 10.3390/s25144355

**Published:** 2025-07-11

**Authors:** Yuanyuan Chai, Furong Zhang, Donglin Jiang, Liying Shao, Jing Wang, Jing Li

**Affiliations:** 1 School of Engineering, Changchun Normal University, Changchun 130032, China; chaiyuanyuancc@163.com (Y.C.); jiangdonglin@ccsfu.edu.cn (D.J.); shaoliying@ccsfu.edu.cn (L.S.); anaelkitty0306@163.com (J.W.); jxazxs123456@163.com (J.L.); 2 School of Electrical and Information Engineering, Jilin Engineering Normal University, Changchun 130052, China

**Keywords:** zeroing neural network, iterative learning control, initial condition algorithm

## Abstract

In this paper, an iterative learning control without resetting conditions based on a finite-time zeroing neural network (NRCILC-FTZNN) is designed for trajectory tracking of a robotic manipulator operating under external disturbances and executing repetitive tasks. A finite-time zeroing neural network (FTZNN) is developed to eliminate external disturbances and enhance convergence. Furthermore, an iterative learning control without resetting conditions based on the FTZNN is proposed to automatically provide the initial state value in each iteration, thereby eliminating the need for reset conditions. The trajectory-tracking errors, measured by the mean absolute error (MAE), are reduced by 46.89% and 63.29% compared to other schemes. Furthermore, the tracking errors of the proposed NRCILC-FTZNN method converge to zero in fewer iterations than those of the other methods. Simulation results demonstrate the convergence of the robotic manipulator system under disturbances to confirm the effectiveness of NRCILC-FTZNN scheme.

## 1. Introduction

Research on robotic manipulators was advanced steadily with the development of automation and intelligent systems [1,2,3,4]. With the advancement of research, numerous control methods for robotic manipulator system were proposed [5,6,7,8]. These methods have enabled robotic manipulators to achieve reliable task execution in complex and uncertain environments.

So far, the zeroing neural network (ZNN) has been regarded as an algorithm characterized by convergence properties [9,10,11]. The ZNN was able to avoid the errors commonly encountered in conventional gradient neural networks and has gained attention over the past two decades. Moreover, the ZNN was recently applied to various control tasks aimed at eliminating uncertainties [12,13,14]. By designing a nonlinear activation function and introducing an integral term, a noise-tolerant ZNN with finite-time convergence was proposed for trajectory tracking [14]. Simulation results confirmed that this noise-tolerant ZNN achieved stable solutions within finite time under uncertainties. Additionally, a novel ZNN, referred to as ST-ZNN, was developed by incorporating the super-twisting (ST) algorithm to handle external disturbances while ensuring finite-time convergence [15]. The ST-ZNN demonstrated improvements in finite-time trajectory-tracking accuracy and disturbance rejection capabilities for parallel robotic manipulator systems. Furthermore, two robust finite-time ZNN (RFTZNN) variants based on stationary and non-stationary parameters with sign-bi-power activation functions were designed to eliminate uncertainties [16]. Thus, the core of the ZNN was to construct an error function that asymptotically approached zero.

The robotic manipulators were widely employed to perform repetitive tasks [17,18]. The iterative learning control (ILC) was aimed at enhancing the performance of a controlled system by leveraging information from previous executions, which enabled the system to improve progressively with each iteration [19,20]. For tracking control of robotic manipulators, a novel proportional-derivative iterative second-order neural network learning control (PDISN) method was proposed [21]. The tracking errors of joints l and 2 were recorded to be approximately $1.2*10^{-7}$ rad and $10^{-7}$ rad, respectively. In addition, an ILC approach was developed to jointly learn model parameters based on the interoceptive sensors [22]. The ILC outperformed the baseline by achieving a threefold reduction in residual vibration. Thus, the ILC was demonstrated to be particularly effective in trajectory tracking for robotic manipulators. Numerous studies on ILC were conducted under the assumption of identical initial conditions. These approaches were typically dependent on the desired state and input, which were not always available beforehand, thereby limiting their practical applicability. Notably, traditional ILC frameworks required the system to start each iteration from the same initial condition. Unlike repetitive control, which reused the terminal condition of the previous cycle as the initial condition of the current one, some ILC algorithms removed the requirement for identical initial conditions. For varying initial states, an ILC framework was proposed to learn from a virtual cycle constructed based on historical data [23]. Furthermore, the ILC was designed with distributed initial-state learning, which eliminated the need to fix the initial value at the start of each iteration [24]. Therefore, the limitations imposed by initial conditions were eliminated through the implementation of the ILC algorithm without resetting conditions.

Building upon the considerations outlined above, an innovative finite-time activation function of the zeroing neural network (FTZNN) is developed in this paper. An ILC without resetting conditions based on the FTZNN (NRCILC-FTZNN) is proposed for a robotic manipulator under external disturbances. The proposed NRCILC-FTZNN is introduced as a novel framework that enhances convergence in repetitive tracking tasks. The main contributions of this paper are summarized as follows:

- A novel FTZNN is introduced to reduce external disturbances and enhance the convergence of the system.
- An ILC without resetting conditions is proposed, which automatically provides the initial state value in each iteration, thereby eliminating the need for reset conditions.
- The convergence of the system is theoretically proven. Moreover, trajectory-tracking simulations further confirm that the proposed NRCILC-FTZNN achieves rapid convergence compared to other schemes and reduces external disturbances.

This paper is organized as follows: Section 2 presents the dynamic model of robotic manipulator. Section 3 introduces the design of the proposed tracking control strategy. In Section 4, the convergence of the NRCILC-FTZNN sheme is theoretically analyzed. Section 5 evaluates the effectiveness of the proposed algorithm through simulation results. Finally, Section 6 summarizes the achievements and predicts the future work.

## 2. Dynamical Model

In this paper, the dynamic model of robotic manipulator (1) is assumed to be known and used as part of the control design framework. The focus of this paper is not on modeling or analyzing the dynamics themselves, but rather on developing the NRCILC-FTZNN.

The robotic manipulator composed of serially connected rigid links is considered. The motion of the *n*-links manipulator is described by the following dynamic equation:

(1) $$M\left(\theta_{k}\left(t\right)\right){\ddot{\theta }}_{k}\left(t\right)+C\left(\theta_{k}\left(t\right),{\dot{\theta }}_{k}\left(t\right)\right){\dot{\theta }}_{k}\left(t\right)+G\left(\theta_{k}\left(t\right)\right)+d\left(t\right)=\tau_{k}\left(t\right)$$

where *k* is the iteration number; *t* is the time; $\theta_{k}\left(t\right)$, ${\dot{\theta }}_{k}\left(t\right)$, and ${\ddot{\theta }}_{k}\left(t\right)$ denote link position, velocity, and acceleration vectors, respectively; $M(\theta_{k}\left(t\right))$, $C(\theta_{k}\left(t\right),{\dot{\theta }}_{k}\left(t\right))$, and $G(\theta_{k}\left(t\right))$ are the manipulator inertia matrix, centripetal and Coriolis matrix, and gravitational torque, respectively; d(t) is the disturbance torque, for example, it can be measurement noises and friction compensation in control; and $\tau_{k}\left(t\right)$ is the torque input vector.

In addition, $\theta_{d}\left(t\right)$, ${\dot{\theta }}_{d}\left(t\right)$, and ${\ddot{\theta }}_{d}\left(t\right)$ denote the desired link position, velocity, and acceleration vectors, respectively. To monitor the tracking process, the tracking error $e_{k}\left(t\right)$ and its time-derivative ${\dot{e}}_{k}\left(t\right)$ are defined as follows:

(2) $$e_{k}\left(t\right)=\theta_{d}\left(t\right)-\theta_{k}\left(t\right)$$

(3) $${\dot{e}}_{k}\left(t\right)={\dot{\theta }}_{d}\left(t\right)-{\dot{\theta }}_{k}\left(t\right)$$

with $E_{k}\left(t\right)={\left[e_{k}\left(t\right),{\dot{e}}_{k}\left(t\right)\right]}^{T}$.

The following modeling assumptions are used in the development and analysis of the proposed controller.

**Modeling Assumptions 1.** For the purpose of controller synthesis and theoretical analysis, the robotic manipulator is modeled as a rigid serial-link mechanism with *n* degrees of freedom, where joint flexibility, mechanical backlash, and structural compliance are neglected.
**Modeling Assumptions 2.** The systems are assumed to be fully known, with the manipulator inertia matrix, centripetal and Coriolis matrix, and gravitational torque regarded as smooth and continuously differentiable functions of their arguments.
**Modeling Assumptions 3.** Frictional effects, sensor noise, and other higher-order uncertainties are not explicitly modeled; instead, they are encompassed within a bounded disturbance term d(t), which accounts for both internal and external unmodeled effects.
**Modeling Assumptions 4.** It is presumed that accurate measurements of joint positions and velocities are available throughout the control.

Although certain aspects of practical robotic systems are idealized by these assumptions, they are widely adopted in model-based and ILC studies to facilitate analytical tractability while preserving dominant characteristics of system.

The following properties, lemmas, and assumptions are used in the development and analysis of the proposed controller.

**Property 1.**  *Thematrices $M(\theta_{k}\left(t\right))$ and $G(\theta_{k}\left(t\right))$ are bounded and Lipschitz continuous with respect to their arguments, as described below:*

(4) $$0<m_{1}<∥M\left(\theta_{k}\left(t\right)\right)∥<m_{2}$$

(5) $$∥G(\theta_{k}\left(t\right)∥\leq g$$

(6) $$∥M\left(\theta_{k+1}\left(t\right)\right)-M\left(\theta_{k}\left(t\right)\right)∥\leq \alpha_{m}∥\theta_{k+1}\left(t\right)-\theta_{k}\left(t\right)∥$$

(7) $$∥G\left(\theta_{k+1}\left(t\right)\right)-G\left(\theta_{k}\left(t\right)\right)∥\leq \alpha_{g}∥\theta_{k+1}\left(t\right)-\theta_{k}\left(t\right)∥$$

*where $m_{1}$, $m_{2}$, g, $\alpha_{m}$, and $\alpha_{g}$ are positive constant.*

**Property 2.**  *The $C(\theta_{k}\left(t\right),{\dot{\theta }}_{k}\left(t\right))$ is bounded as follows:*

(8) $$∥C\left(\theta_{k}\left(t\right),{\dot{\theta }}_{k}\left(t\right)\right)∥\leq c∥{\dot{\theta }}_{k}\left(t\right)∥$$

*where c is positive constant.*

**Property 3.**  *The i-th element of $C\left(\theta_{k}\left(t\right),{\dot{\theta }}_{k}\left(t\right)\right){\dot{\theta }}_{k}\left(t\right)$∀i∈[1,...,n] is equal to ${\dot{\theta }}_{k}{\left(t\right)}^{T}N_{i}\left(\theta_{k}\left(t\right)\right){\dot{\theta }}_{k}\left(t\right)$, where $N_{i}\left(\theta_{k}\left(t\right)\right)$ is symmetric and continuously differentiable, satisfying the following condition:*

(9) $$∥N_{i}\left(\theta_{k}\left(t\right)\right)∥\leq {\bar{N}}_{i}$$

*where $\exists {\bar{N}}_{i}>0$.*

**Lemma 1**  ([25]). *The $M(\theta_{k}\left(t\right))$ possesses the following property:*

(10) $$∥M^{-1}\left(\theta_{k+1}\left(t\right)\right)-M^{-1}\left(\theta_{k}\left(t\right)\right)∥\leq \alpha_{m}m_{1}^{-2}∥\theta_{k+1}\left(t\right)-\theta_{k}\left(t\right)∥.$$

**Proof of Lemma 1.**  See [25,26]. □

**Lemma 2**  ([27]). The norm of a function (*) over the t∈[0,T] is defined as follows:

(11) $${∥*\left(t\right)∥}_{\lambda }=\sup(e^{-\lambda t}∥*\left(t\right)∥).$$

In addition, let $\Omega \left(t\right)={\left[\Omega_{1}\left(t\right),\Omega_{2}\left(t\right),\ldots \Omega_{n}\left(t\right)\right]}^{T}\in R^{n}$ be defined. Then,

(12) $$e^{-\lambda t}\int_{0}^{t}∥\Omega \left(\delta \right)∥d\delta \leq \frac{1}{\lambda }{∥\Omega \left(t\right)∥}_{\lambda }.$$

**Proof of Lemma 2.**  See [26,27]. □

**Assumption 1.**  *The norms of d(t) and $\theta_{k}\left(t\right)$ are bounded by the positive constants $\alpha_{d}$ and $\alpha_{\theta }$, respectively.*

## 3. Tracking Control Design

**Figure 1:**
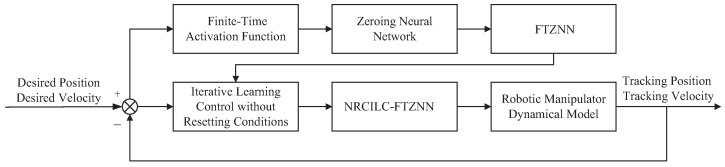
Structure of NRCILC-FTZNN.

This section introduces the evolution formula of the ZNN and proposes a novel activation function. In addition, by integrating the FTZNN, an ILC algorithm without resetting conditions (NRCILC-FTZNN) is proposed as shown in Figure 1.

### 3.1. Finite-Time Zeroing Neural Network

ZNN is a specialized RNN variant that efficiently handles the zero-finding problem [28], it is written as follows:

(13) $$\xi (x_{k}\left(t\right),t)=0$$

where $\xi (x_{k}\left(t\right),t)$ is a function. Therefore, ZNN is designed to determine the exact solution $x_{k}^{*}\left(t\right)$. The corresponding error function is formulated as follows:

(14) $$\Phi \left(Z\left(t\right)\right)=\xi \left(x_{k}^{*}\left(t\right),t\right)-\xi \left(x_{k}\left(t\right),t\right)=0-\xi \left(x_{k}\left(t\right),t\right)$$

where $Z\left(t\right)\in R^{n}$ denotes the state variable; Φ(·) represents an array of activation functions.

For the error function Φ(Z(t)) to asymptotically approach zero, the state variable $x_{k}\left(t\right)$ converges to the $x_{k}^{*}\left(t\right)$. Consequently, the zero-finding problem (13) can be reformulated as follows:

(15) $$\left\{\begin{array}{c} {\dot{x}}_{k}\left(t\right)=u_{k}\left(t\right)\\ y_{k}\left(t\right)=\xi \left(x_{k}\left(t\right),t\right)=-\Phi \left(Z\left(t\right)\right) \end{array}\right.$$

with $u_{k}\left(t\right)$ denoting a control input function that ensures Φ(Z(t)) converges to zero. Hence, the ZNN is proposed as follows:

(16) $$\dot{Z}\left(t\right)=-\eta \Phi \left(Z\left(t\right)\right)$$

where η>0 is a fixed parameter that governs the convergence of $\dot{Z}\left(t\right)$.

**Theorem 1**  ([29]). *Under ZNN, the state variable achieves global exponential convergence to the theoretical solution when the error function satisfies the prescribed conditions.*

**Proof of Theorem 1.**  See [29]. □

By employing an appropriately designed activation function, Φ(·) being a scalar activation function, $\dot{Z}\left(t\right)$ can be driven to converge to zero within a finite amount of time. Accordingly, the element ϕ(·) of the finite-time activation function Φ(·) is defined as follows:

(17) $$\phi \left(z\left(t\right)\right)=\beta_{1}z\left(t\right)+\beta_{2}z{\left(t\right)}^{\frac{q}{p}}$$

where z(t) is the element of $\dot{Z}\left(t\right)$; $\beta_{1}$ and $\beta_{2}$ are positive constant; *q* and *p* are positive odd integers that satisfy the condition p>q.

When the finite-time activation function (17) and the input $e_{k}\left(t\right)$ are considered, the evolution Formula (16) with η=1 can be rewritten as follows:

(18) $${\dot{e}}_{k}\left(t\right)=-\beta_{1}e_{k}\left(t\right)-\beta_{2}e_{k}{\left(t\right)}^{\frac{q}{p}}.$$

The control input function based on finite-time activation function of ZNN is constructed as follows:

(19) $$u_{k}\left(t\right)={\dot{e}}_{k}\left(t\right)+\beta_{1}e_{k}\left(t\right)+\beta_{2}e_{k}{\left(t\right)}^{\frac{q}{p}}.$$

### 3.2. Iterative Learning Control Without Restting Condition

Given the robotic manipulator described by (1), and under the condition that the assumption is satisfied, the following control law is applied for any initial joint position $\theta_{k}\left(0\right)$ and initial joint velocity ${\dot{\theta }}_{k}\left(0\right)$:

(20) $$\tau_{k+1}\left(t\right)=\tau_{k}\left(t\right)+M\left(\theta_{k}\left(t\right)\right)\gamma u_{k}\left(t\right)$$

with

(21) $$\left\{\begin{array}{c} e_{k+1}\left(0\right)=e_{k}\left(0\right)\\ {\dot{e}}_{k+1}\left(0\right)={\dot{e}}_{k}\left(0\right)-\gamma e_{k}\left(0\right). \end{array}\right.$$

where γ is a symmetric, positive, and definite matrix.

## 4. Theoretical Analysis

In this section, convergence analysis of the system (1) is conducted.

**Theorem 2.**  *If $∥I_{n}-\gamma ∥<1$, the system (1) utilizing controller* (20) *is asymptotically stable. Therefore,*

(22) $$\lim_{k\to \infty }\left(\theta_{d}\left(t\right)-\theta_{k}\left(t\right)\right)=\lim_{k\to \infty }\left({\dot{\theta }}_{d}\left(t\right)-{\dot{\theta }}_{k}\left(t\right)\right)=0$$

*where $I_{n}$ is identity matrix.*

**Proof of Theorem 1.**  According to (1), it can be expressed as follows:

(23) $$\left\{\begin{array}{c} {\dot{\theta }}_{1k}\left(t\right)=\theta_{2k}\left(t\right)\\ \begin{array}{cc} {\dot{\theta }}_{2k}\left(t\right) & =M^{-1}\left(\theta_{1k}\left(t\right)\right)\left[-C\left(\theta_{1k}\left(t\right),{\dot{\theta }}_{1k}\left(t\right)\right){\dot{\theta }}_{1k}\left(t\right)-G\left(\theta_{1k}\left(t\right)\right)-d\left(t\right)+\tau_{k}\left(t\right)\right] \end{array} \end{array}\right.$$

where $\theta_{1k}\left(t\right)$ and $\theta_{2k}\left(t\right)$ are link position and velocity vectors, respectively. Thus, letting $\Theta_{k}\left(t\right)={\left[\theta_{1k}\left(t\right),\theta_{2k}\left(t\right)\right]}^{T}$, (23) is written as:

(24) $${\dot{\Theta }}_{k}\left(t\right)=B\Theta_{k}\left(t\right)+D\tau_{k}\left(t\right)+F_{k}\left(t\right)$$

where $B=\left[\begin{array}{cc} 0_{n} & I_{n}\\ 0_{n} & 0_{n} \end{array}\right]$, $D=\left[\begin{array}{c} 0_{n}\\ M^{-1}\left(\theta_{k}\left(t\right)\right) \end{array}\right]$, and $F_{k}\left(t\right)=\left[\begin{array}{c} 0_{n}\\ f_{k}\left(t\right) \end{array}\right]$ with

(25) $$f_{k}\left(t\right)=-M^{-1}\left(\theta_{k}\left(t\right)\right)\left[C\left(\theta_{k}\left(t\right),{\dot{\theta }}_{k}\left(t\right)\right){\dot{\theta }}_{k}\left(t\right)+G\left(\theta_{k}\left(t\right)\right)+d\left(t\right)\right].$$

From (24), the general solution $\Theta_{k}\left(t\right)$ is given by the following:

(26) $$\Theta_{k}\left(t\right)=e^{At}\Theta_{k}\left(0\right)+\int_{0}^{t}e^{A(t-\delta )}D\tau_{k}\left(\delta \right)d\delta +\int_{0}^{t}e^{A(t-\delta )}F_{k}\left(\delta \right)d\delta .$$

where $e^{At}$ represents the state transition matrix of the unforced isolated system. Thus, $\Theta_{k+1}\left(t\right)$ can be derived.

By subtracting $\Theta_{k}\left(t\right)$ from $\Theta_{k+1}\left(t\right)$ and according to (20), the expression is derived as follows:

(27) $$\begin{array}{cc} \Theta_{k+1}\left(t\right)-\Theta_{k}\left(t\right)= & e^{At}\Gamma e_{k}\left(0\right)+\int_{0}^{t}e^{A(t-\delta )}\Gamma {\dot{e}}_{k}\left(\delta \right)d\delta \\ \; & +\int_{0}^{t}e^{A(t-\delta )}\Gamma \left(\beta_{1}e_{k}\left(\delta \right)+\beta_{2}e_{k}{\left(\delta \right)}^{\frac{q}{p}}\right)d\delta \\ \; & +\int_{0}^{t}e^{A(t-\delta )}\left(F_{k+1}\left(\delta \right)-F_{k}\left(\delta \right)\right)d\delta \end{array}$$

with $DM(\theta_{k}\left(t\right))\gamma =\Gamma$, thus, $\Gamma ={\left[0,\gamma \right]}^{T}$.

By integrating the term ${\dot{e}}_{k}\left(t\right)$ by parts, the following expression is obtained:

(28) $$\begin{array}{cc} \Theta_{k+1}\left(t\right)-\Theta_{k}\left(t\right)= & \Gamma e_{k}\left(t\right)+\int_{0}^{t}e^{A(t-\delta )}\Gamma Ae_{k}\left(\delta \right)d\delta \\ \; & +\int_{0}^{t}e^{A(t-\delta )}\Gamma \left(\beta_{1}e_{k}\left(\delta \right)+\beta_{2}e_{k}{\left(\delta \right)}^{\frac{q}{p}}\right)d\delta \\ \; & +\int_{0}^{t}e^{A(t-\delta )}\left(F_{k+1}\left(\delta \right)-F_{k}\left(\delta \right)\right)d\delta . \end{array}$$

(28) adds and subtracts $\theta_{d}\left(t\right)$ and ${\dot{\theta }}_{d}\left(t\right)$, which is generalized as follows:

(29) $$\begin{array}{cc} E_{k+1}\left(t\right)= & E_{k}\left(t\right)-\Gamma e_{k}\left(t\right)-\int_{0}^{t}e^{A(t-\delta )}\Gamma Ae_{k}\left(\delta \right)d\delta \\ \; & -\int_{0}^{t}e^{A(t-\delta )}\Gamma \left(\beta_{1}e_{k}\left(\delta \right)+\beta_{2}e_{k}{\left(\delta \right)}^{\frac{q}{p}}\right)d\delta \\ \; & -\int_{0}^{t}e^{A(t-\delta )}\left(F_{k+1}\left(\delta \right)-F_{k}\left(\delta \right)\right)d\delta . \end{array}$$

Taking the norm of both sides of (29) and applying standard norm properties yields the following:

(30) $$\begin{array}{cc} ∥E_{k+1}\left(t\right)∥\leq & ∥E_{k}\left(t\right)-\Gamma e_{k}\left(t\right)∥+\int_{0}^{t}∥e^{A(t-\delta )}∥∥\Gamma \beta_{2}∥∥e_{k}{\left(\delta \right)∥}^{\frac{q}{p}}d\delta \\ \; & +\int_{0}^{t}∥e^{A(t-\delta )}∥(∥\Gamma A∥+∥\Gamma \beta_{1}∥)∥e_{k}\left(\delta \right)∥d\delta \\ \; & +\int_{0}^{t}∥e^{A(t-\delta )}∥∥F_{k+1}\left(\delta \right)-F_{k}\left(\delta \right)∥d\delta \end{array}$$

Due to $E_{k}\left(t\right)={\left[e_{k}\left(t\right),{\dot{e}}_{k}\left(t\right)\right]}^{T}$, it is clear that $∥e_{k}\left(t\right)∥\leq ∥E_{k}\left(t\right)∥$, $∥e_{k}{\left(t\right)∥}^{\frac{q}{p}}\leq ∥E_{k}\left(t\right)∥$, $∥{\dot{e}}_{k}\left(t\right)∥\leq ∥E_{k}\left(t\right)∥$, and $∥E_{k}\left(t\right)-\Gamma e_{k}\left(t\right)∥\leq ∥I^{2}-\Gamma ∥∥E_{k}\left(t\right)∥$ with $I^{2}={\left[I_{n},I_{n}\right]}^{T}$. Therefore, (30) can be expressed as follows:

(31) $$\begin{array}{cc} ∥E_{k+1}\left(t\right)∥\leq & ∥I^{2}-\Gamma ∥∥E_{k}\left(t\right)∥+\int_{0}^{t}∥e^{A(t-\delta )}∥∥F_{k+1}\left(\delta \right)-F_{k}\left(\delta \right)∥d\delta \\ \; & +\int_{0}^{t}∥e^{A(t-\delta )}∥(∥\Gamma A∥+∥\Gamma \beta_{1}∥+∥\Gamma \beta_{2}∥)∥E_{k}\left(\delta \right)∥d\delta \end{array}$$

According to (25), $f_{k+1}\left(t\right)-f_{k}\left(t\right)$ can be obtained as follows:

(32) $$\begin{array}{cc} f_{k+1}\left(t\right)-f_{k}\left(t\right)= & -(M^{-1}\left(\theta_{k+1}\left(t\right)\right)-M^{-1}\left(\theta_{k}\left(t\right)\right))d\left(t\right)\\ \; & -\left(M^{-1}\left(\theta_{k+1}\left(t\right)\right)-M^{-1}\left(\theta_{k}\left(t\right)\right)\right)G\left(\theta_{k}\left(t\right)\right)\\ \; & -\left(M^{-1}\left(\theta_{k+1}\left(t\right)\right)-M^{-1}\left(\theta_{k}\left(t\right)\right)\right)C\left(\theta_{k}\left(t\right),{\dot{\theta }}_{k}\left(t\right)\right){\dot{\theta }}_{k}\left(t\right)\\ \; & -M^{-1}\left(\theta_{k+1}\left(t\right)\right)\left(G\left(\theta_{k+1}\left(t\right)\right)-G\left(\theta_{k}\left(t\right)\right)\right)\\ \; & -M^{-1}\left(\theta_{k+1}\left(t\right)\right)(C\left(\theta_{k+1}\left(t\right),{\dot{\theta }}_{k+1}\left(t\right)\right){\dot{\theta }}_{k+1}\left(t\right)\\ \; & -C\left(\theta_{k}\left(t\right),{\dot{\theta }}_{k}\left(t\right)\right){\dot{\theta }}_{k}\left(t\right)). \end{array}$$

Using Property 3, it follows that ∃ω>0 such that

(33) $$∥\frac{\partial }{\partial {\dot{\theta }}_{k}\left(t\right)}\left(C\left(\theta_{k}\left(t\right),{\dot{\theta }}_{k}\left(t\right)\right){\dot{\theta }}_{k}\left(t\right)\right)∥\leq \omega$$

with

(34) $$\frac{\partial }{\partial {\dot{\theta }}_{k}\left(t\right)}\left(C\left(\theta_{k}\left(t\right),{\dot{\theta }}_{k}\left(t\right)\right){\dot{\theta }}_{k}\left(t\right)\right)=2{\left[{\dot{\theta }}_{k}{\left(t\right)}^{T}N_{1}\left(\theta_{k}\left(t\right)\right),...,{\dot{\theta }}_{k}{\left(t\right)}^{T}N_{n}\left(\theta_{k}\left(t\right)\right)\right]}^{T}$$

According to Property 1, Property 2, Lemma 1, Assumption 1, and (33), the norm of (32) can be generalized as follows:

(35) $$∥f_{k+1}\left(t\right)-f_{k}\left(t\right)∥\leq \rho ∥E_{k+1}\left(t\right)-E_{k}\left(t\right)∥$$

where $\rho =\alpha_{m}m_{1}^{-2}\left(c\alpha_{\theta }^{2}+\alpha_{d}+g\right)+m_{1}^{-1}\left(\alpha_{g}+\omega \right)$.

From (30) and (35), the expression is given as follows:

(36) $$\begin{array}{cc} ∥E_{k+1}\left(t\right)∥\leq & ∥I^{2}-\Gamma ∥∥E_{k}\left(t\right)∥+\psi \xi \int_{0}^{t}∥E_{k}\left(\delta \right)∥d\delta \\ \; & +\psi \rho \int_{0}^{t}∥E_{k+1}\left(\delta \right)-E_{k}\left(\delta \right)∥d\delta . \end{array}$$

where $\psi :=\sup_{t,s\in [0,T]}∥e^{A(t-\delta )}∥$ and $\xi =∥\Gamma A∥+∥\Gamma \beta_{1}∥+∥\Gamma \beta_{2}∥$.

Multiplying both sides of (36) by $e^{-˘t}$ and applying Lemma 2 yields the following expression:

(37) $$∥E_{k+1}{\left(t\right)∥}_{\lambda }\leq \frac{∥I^{2}-\Gamma ∥+\frac{\psi \xi +\psi \rho }{\lambda }}{1-\frac{\psi \rho }{\lambda }}{∥E_{k}\left(t\right)∥}_{\lambda }.$$

By condition $∥I^{2}-\Gamma ∥<1$, thus $∥I_{n}-\gamma ∥<1$, it follows that there exists λ(λ>0) large enough such that for ∀t∈[0,T]

(38) $$\frac{∥I^{2}-\Gamma ∥+\frac{\psi \xi +\psi \rho }{\lambda }}{1-\frac{\psi \rho }{\lambda }}=:\chi <1.$$

Thus, the following is obtained:

(39) $$∥E_{k+1}{\left(t\right)∥}_{\lambda }\leq \chi {∥E_{k}\left(t\right)∥}_{\lambda }.$$

Form (38) and (39), it can be obtained as follows:

(40) $$\lim_{k\to \infty }{∥E_{k}\left(t\right)∥}_{\lambda }=0.$$

Thus,

(41) $$\lim_{k\to \infty }\left(\theta_{d}\left(t\right)-\theta_{k}\left(t\right)\right)=\lim_{k\to \infty }\left({\dot{\theta }}_{d}\left(t\right)-{\dot{\theta }}_{k}\left(t\right)\right)=0$$

Therefore, (41) is verified, which completes the proof. □

## 5. Simulation and Discussion

**Figure 2:**
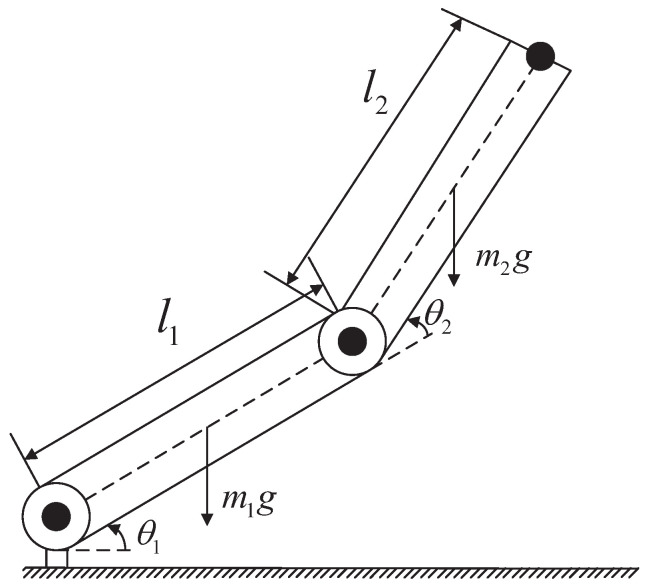
The robotic manipulator.

The NRCILC-FTZNN algorithm is implemented in MATLAB. Moreover, three illustrative examples are provided to validate the characteristics and effectiveness of the NRCILC-FTZNN in trajectory tracking. The robotic manipulator is depicted in Figure 2. Moreover, the manipulator link weight is set to 2 kg and the link length to 0.6 m [30,31]. The model (1) with the elements of $M(\theta_{k}\left(t\right))$, $C(\theta_{k}\left(t\right),{\dot{\theta }}_{k}\left(t\right))$, and $G(\theta_{k}\left(t\right))$ are given by the following:

(42) $$\begin{array}{cc} \; & m_{11}=m_{1}l_{c1}^{2}+m_{2}l_{1}^{2}+m_{2}l_{c2}^{2}+2m_{2}l_{1}l_{c2}\cos\left(\theta_{2}\left(t\right)\right)+I_{1}+I_{2}\\ \; & m_{12}=m_{2}l_{c2}^{2}+m_{2}l_{1}l_{c2}\cos\left(\theta_{2}\left(t\right)\right)+I_{2}\\ \; & m_{21}=m_{12}\\ \; & m_{22}=m_{2}l_{c2}^{2}+I_{2}\\ \; & M\left(\theta \left(t\right)\right)=\left[m_{11}\;m_{12};m_{21}\;m_{22}\right]\\ \; & c_{11}=-m_{2}l_{1}l_{c2}\sin\left(\theta_{2}\left(t\right)\right){\dot{\theta }}_{2}\left(t\right)\\ \; & c_{12}=-m_{2}l_{1}l_{c2}\sin\left(\theta_{2}\left(t\right)\right)\left({\dot{\theta }}_{1}\left(t\right)+{\dot{\theta }}_{2}\left(t\right)\right)\\ \; & c_{21}=m_{2}l_{1}l_{c2}\sin\left(\theta_{2}\left(t\right)\right){\dot{\theta }}_{1}\left(t\right)\\ \; & c_{22}=0\\ \; & C\left(\theta \left(t\right),\dot{\theta }\left(t\right)\right)=\left[c_{11}\;c_{12};c_{21}\;c_{22}\right]\\ \; & g_{1}=m_{2}l_{c2}g\sin\left(\theta_{1}\left(t\right)+\theta_{2}\left(t\right)\right)+\left(m_{1}l_{c1}+m_{2}l_{1}\right)g\sin\left(\theta_{1}\left(t\right)\right)\\ \; & g_{2}=m_{2}l_{c2}g\sin\left(\theta_{1}\left(t\right)+\theta_{2}\left(t\right)\right)\\ \; & G\left(\theta \left(t\right)\right)=\left[g_{1};g_{2}\right] \end{array}$$

where $m_{1}$ and $m_{2}$ are masses, $l_{1}$ and $l_{2}$ are lengths, $l_{c1}$ and $l_{c2}$ are the distance between the center of mass of the joint and the corresponding link, $I_{1}$ and $I_{2}$ are moment of inertia, and *g* is gravitational acceleration. The robot parameters are set as $m_{1}=m_{2}=2$ kg, $l_{1}=l_{2}=0.6$ m, $l_{c1}=l_{c2}=0.4$ m, $I_{1}=I_{2}=0.1\;\mathrm{kg}\;m^{2}/\mathrm{rad}$, and g=9.81.

This section focuses on investigating the simulation and effectiveness of the NRCILC-FTZNN applied to robotic manipulator. To establish a rigorous and quantitative evaluation framework, the root mean square error (RMSE), mean absolute error (MAE), and standard deviation (SD) are utilized. These metrics are defined as follows:

(43) $$\mathrm{RMSE}=\sqrt{\frac{1}{X}\sum_{k=1}^{X}{\left(v_{t}-v_{o}\right)}^{2}}$$

(44) $$\mathrm{MAE}=\frac{1}{X}\sum_{k=1}^{X}\left|v_{t}-v_{o}\right|$$

(45) $$\mathrm{SD}=\sqrt{\frac{1}{X}\sum_{k=1}^{X}{\left(v_{o}-v_{a}\right)}^{2}}$$

where *X* is number of data; and $v_{t}$, $v_{o}$, and $v_{a}$ are the true value, observation value, and average of observation value, respectively.

### 5.1. Comparative Simulation with Robust Adaptive Proportional-Derivative Control

This section compares the NRCILC-FTZNN framework with robust adaptive proportional-derivative (RAPD) control. Additionally, the tracking controller of RAPD is defined as follows:

(46) $$\begin{array}{cc} \tau = & -\left(K_{p1}+K_{p2}K_{p}\left(e\left(t\right)\right)\right)e\left(t\right)-\left(K_{v1}+K_{v2}K_{v}\left(\dot{e}\left(t\right)\right)\right)\dot{e}\left(t\right)+\Psi \left(\theta \left(t\right),\dot{\theta }\left(t\right),{\dot{\theta }}_{r}\left(t\right),{\ddot{\theta }}_{r}\right)\left(t\right)\hat{P}\\ \; & -d(t)sign(\mu ) \end{array}$$

where $K_{p}\left(e\left(t\right)\right)=diag\left(\frac{1}{p_{1}+\left|e_{1}\right|},\frac{1}{p_{2}+\left|e_{2}\right|},\ldots ,\frac{1}{p_{n}+\left|e_{n}\right|}\right)$,$K_{v}\left(\dot{e}\left(t\right)\right)=diag\left(\frac{1}{v_{1}+\left|{\dot{e}}_{1}\right|},\frac{1}{v_{2}+\left|{\dot{e}}_{2}\right|},\ldots ,\frac{1}{v_{n}+\left|{\dot{e}}_{n}\right|}\right)$.

Moreover, the parameter estimation law for $\hat{P}$ is chosen as follows:

(47) $$\dot{\hat{P}}=-Q\Psi^{T}\left(\theta \left(t\right),\dot{\theta }\left(t\right),{\dot{\theta }}_{r}\left(t\right),{\ddot{\theta }}_{r}\left(t\right)\right)\mu$$

where $\mu =\dot{e}\left(t\right)+\sigma e\left(t\right)$, ${\dot{\theta }}_{r}\left(t\right)={\dot{\theta }}_{d}\left(t\right)-\sigma e\left(t\right)$, Q is a positive, definite, and symmetric matrix.

The parameters are set as $K_{p1}=diag\left(190,190\right)$, $K_{p2}=diag\left(150,150\right)$, $K_{v1}=diag\left(180,180\right)$, $K_{v2}=diag\left(150,150\right)$, σ=5, $Q=5I_{n}$, $p_{i}=v_{i}=1,i=1,2,\ldots ,n$.

**Figure 3:**
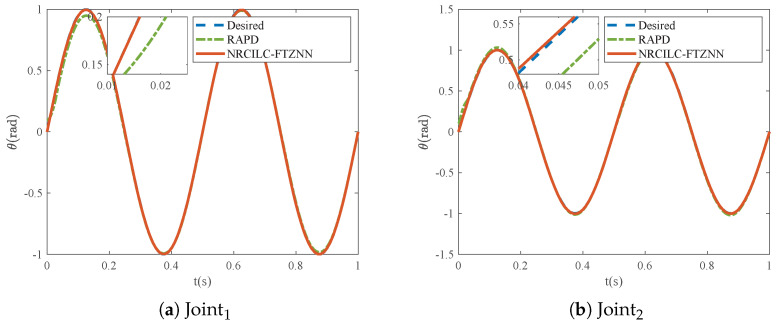
Tracking trajectories of robotic manipulator based on RAPD and NRCILC-FTZNN.

**Figure 4:**
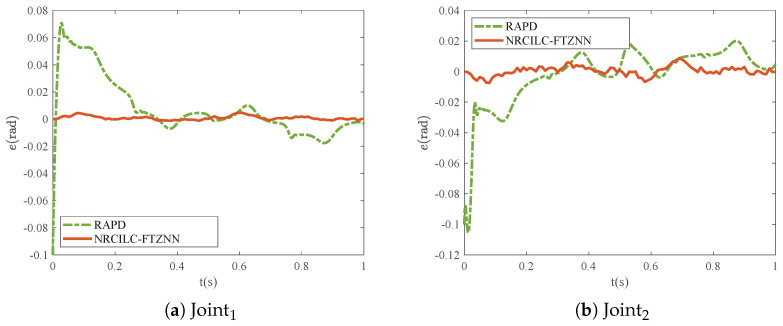
Tracking errors of robotic manipulator based on RAPD and NRCILC-FTZNN.

As shown in Figure 3, the tracking trajectories generated by the RAPD and NRCILC-FTZNN are illustrated. Both methods exhibit convergence of tracking trajectories to the desired trajectories. Furthermore, the comparison of the tracking errors of the RAPD and NRCILC-FTZNN is shown in Figure 4. The tracking error of the NRCILC-FTZNN is smaller than those of the RAPD, which indicates superior tracking performance.

**Figure 5:**
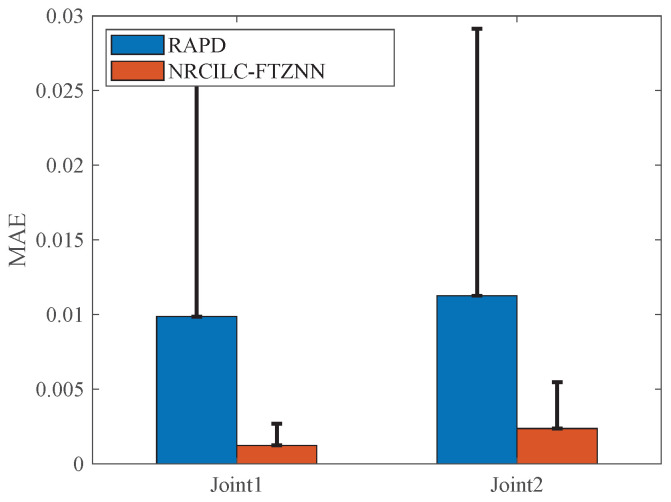
The MAE of RAPD and NRCILC-FTZNN. (The error bar is SD).

The comparisons of the RMSE, MAE, and SD of the RAPD and NRCILC-FTZNN are shown in Table 1 and Figure 5. The RMSE values of the NRCILC-FTZNN are 0.0017 and 0.0031, both smaller than those of RAPD. A smaller RMSE indicates that the NRCILC-FTZNN achieves higher tracking accuracy. Additionally, The average trajectory-tracking error of ${\mathrm{Joint}}_{1}$ and ${\mathrm{Joint}}_{2}$, measured by MAE, is reduced by 82.94% compared to RAPD. The MAE and SD of the NRCILC-FTZNN are smaller than RAPD. The smaller MAE demonstrates that the NRCILC-FTZNN maintains stable tracking performance. The lower SD indicates reduced error fluctuations, further confirming excellent stability.

### 5.2. System Simulation with Different Schemes

In this section, various activation functions of ZNN are introduced to highlight the advantages of the NRCILC-FTZNN framework, including the linear activation function (NRCILC-LZNN) and power activation function (NRCILC-PZNN). Additionally, the element form of activation functions for the NRCILC-LZNN and NRCILC-PZNN are defined as follows:

(48) $$\phi \left(z\left(t\right)\right)=\beta_{1}z\left(t\right)$$

(49) $$\phi \left(z\left(t\right)\right)=\beta_{2}z{\left(t\right)}^{\frac{q}{p}}$$

Additionally, the relevant parameters are set as $\beta_{1}=3$, $\beta_{2}=3$, γ=0.25, and $d\left(t\right)=\frac{1}{6}\pi +\frac{1}{6}\pi t+\frac{1}{6}\pi \mathrm{rand}\left(1\right)$.

**Figure 6:**
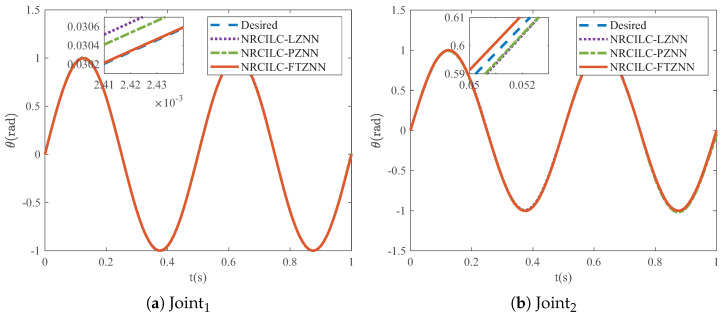
Tracking trajectories of robotic manipulator by three different schemes.

**Figure 7:**
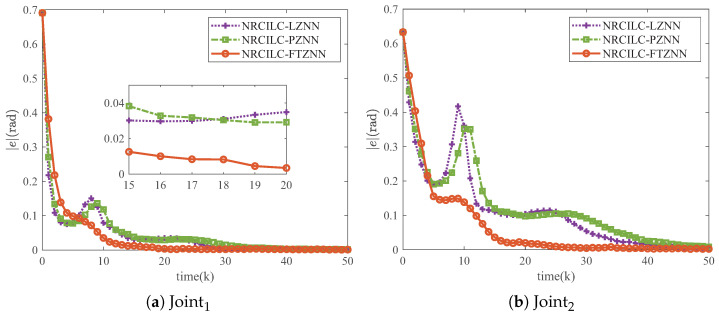
Tracking errors of robotic manipulator by three different schemes in iterations.

As shown in Figure 6, the tracking trajectories generated by the three schemes are illustrated, with the blue dashed lines representing the desired trajectories. It can be observed that the tracking trajectories of the three schemes converge to the desired trajectories. Furthermore, Figure 7 illustrates the mean absolute tracking error per iteration for the three schemes. The tracking errors of the NRCILC-FTZNN converge to zero in fewer iterations compared to the other schemes, which demonstrates the rapid convergence.

The tracking error of NRCILC-FTZNN converges to zero with 10 fewer iterations compared to other schemes, which proves its fast convergence.

**Figure 8:**
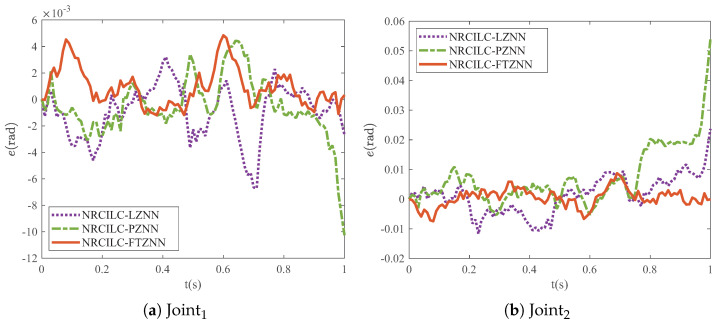
Final tracking errors of robotic manipulator by three different schemes.

A comparison of the final tracking errors of different schemes is shown in Figure 8. The tracking error of the NRCILC-FTZNN is smaller than those of the other schemes, which indicates superior tracking performance. The tracking errors of the NRCILC-FTZNN remain consistently within ±0.005 rad and ±0.01 rad. Furthermore, the error magnitude is 0.005 rad smaller than other schemes, which demonstrates the asymptotic stability of the NRCILC-FTZNN.

As shown in Table 2, under the conditions $\beta_{1}=3$, $\beta_{2}=3$, and $d\left(t\right)=\frac{1}{6}\pi +\frac{1}{6}\pi t+\frac{1}{6}\pi \mathrm{rand}\left(1\right)$, the RMSE of the NRCILC-FTZNN scheme are 0.0017 and 0.0031, which are the lowest among the three schemes. It demonstrates that the higher accuracy of the proposed tracking control scheme in trajectory tracking.

**Figure 9:**
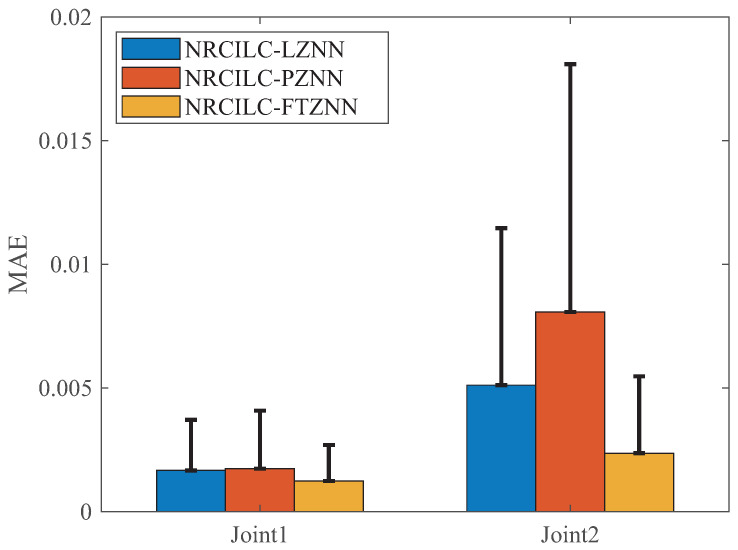
The MAE of three different schemes. (The error bar is SD).

The comparison of MAE and SD for the three schemes is shown in Figure 9. The MAE evaluates the average deviation between the tracking and desired trajectories, while the SD measures the stability of the trajectory tracking. As shown in the figure, both the MAE and SD of the NRCILC-FTZNN are smaller than those of the other schemes. The average trajectory-tracking error of ${\mathrm{Joint}}_{1}$ and ${\mathrm{Joint}}_{2}$, calculated by MAE, are reduced by 46.89% and 63.29% compared to other methods, respectively. A smaller MAE value indicates higher accuracy in trajectory tracking, while a smaller SD value suggests reduced trajectory fluctuation and improved stability.

**Figure 10:**
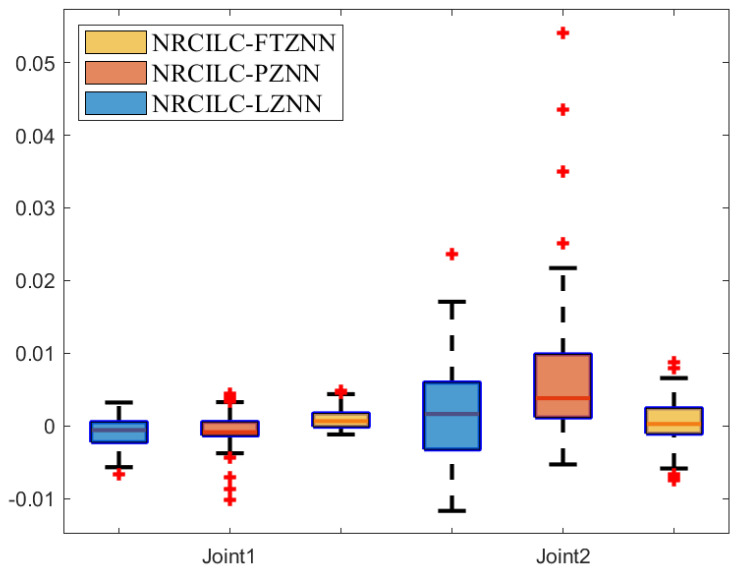
The error box plot of three different schemes.

The box plot, a powerful tool for data visualization, is used to reflect the central tendency, dispersion, and outliers of the error data in Figure 10. Specifically, the median tracking error of the NRCILC-FTZNN is close to zero, which demonstrates the effectiveness in centering the tracking error around zero. Furthermore, the interquartile range as indicated by the length of the box is narrower than that of the other schemes, which reflects the reduced tracking error dispersion. Thus, the NRCILC-FTZNN provides more stable tracking performance.Moreover, the shorter whiskers indicate fewer extreme values. Therefore, the tracking error is centered around zero with low dispersion and a concentrated data distribution. The proposed scheme demonstrates excellent performance in trajectory tracking.

### 5.3. System Simulation with Different Disturbances

This section demonstrates that the NRCILC-FTZNN effectively performs trajectory tracking under different disturbances, including constant disturbance $d\left(t\right)=\frac{1}{6}\pi$, linear disturbance $d\left(t\right)=\frac{1}{6}\pi t$, random disturbance $d\left(t\right)=\frac{1}{6}\pi \mathrm{rand}\left(1\right)$, and mixed disturbance $d\left(t\right)=\frac{1}{6}\pi +\frac{1}{6}\pi t+\frac{1}{6}\pi \mathrm{rand}\left(1\right)$. Additionally, the parameters are set as $\beta_{1}=3$, $\beta_{2}=3$, and γ=0.25.

**Figure 11:**
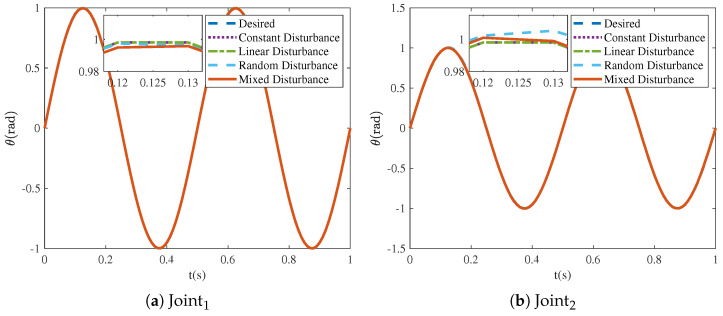
Tracking trajectories of robotic manipulator with four different disturbances.

**Figure 12:**
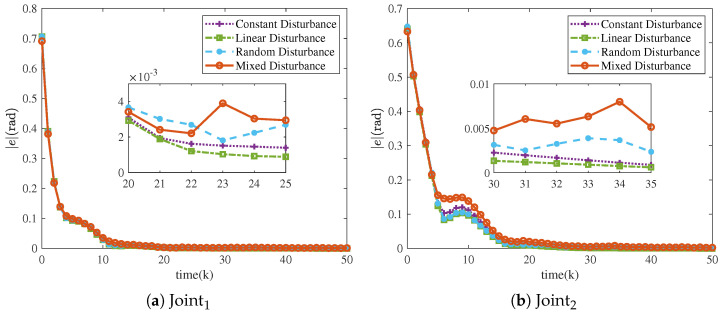
Tracking errors of robotic manipulator with four different disturbances in iterations.

The trajectory tracking under four different types of disturbances is illustrated in Figure 11. The NRCILC-FTZNN is observed to maintain stable tracking performance under various disturbances. Moreover, Figure 12 illustrates the mean absolute error over iterations under different disturbances. The tracking errors converge within 0.005 rad and 0.01 rad, which eliminates external disturbances. The simulation results demonstrate that the NRCILC-FTZNN effectively suppresses disturbances and maintains high tracking accuracy.

**Figure 13:**
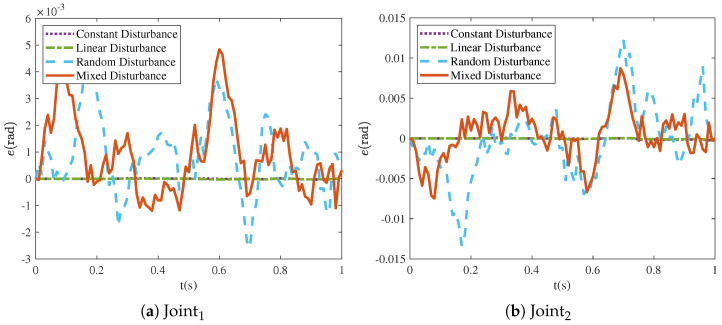
Final tracking errors of robotic manipulator with four different disturbances.

A comparison of the final tracking errors of the NRCILC-FTZNN with different disturbances is shown in Figure 13. The error magnitude of ${\mathrm{Joint}}_{1}$ and ${\mathrm{Joint}}_{2}$ under constant and linear disturbances are 0.006 rad and 0.015 rad smaller than random and mixed disturbances. The system exhibits better performance under constant and linear disturbance compared to random and mixed disturbance, as the models for constant and linear disturbance are relatively simple and more easily compensated for.

**Figure 14:**
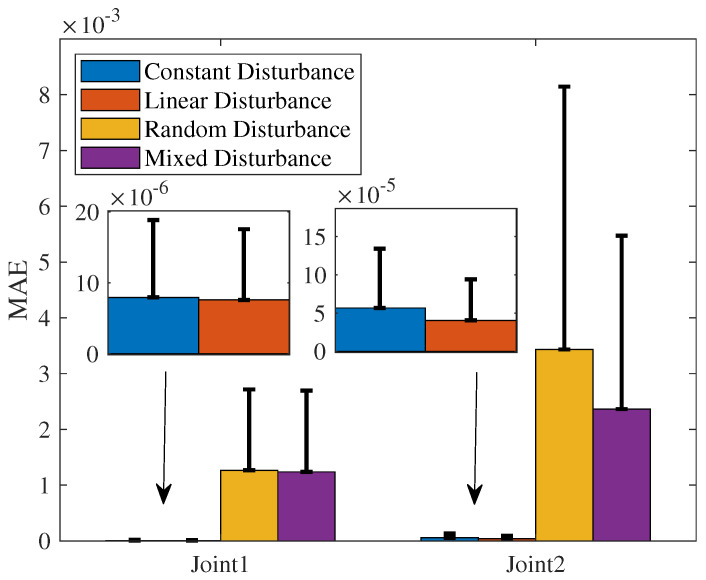
The MAE of NRCILC-FTZNN with four different disturbances. (The error bar is SD).

The comparisons of the RMSE, MAE, and SD of the NRCILC-FTZNN under four different disturbances are shown in Table 2 and Figure 14. It is evident that the RMSE of constant and linear disturbances ranges from 0.0001 to 0.0009, which is smaller than that of random and mixed disturbances. A smaller RMSE indicates that the NRCILC-FTZNN achieves higher tracking accuracy under constant and linear disturbances with a reduced frequency of large errors. Additionally, the MAE and SD of constant and linear disturbances are smaller than those of random and mixed disturbances. The smaller MAE demonstrates that the NRCILC-FTZNN maintains stability in tracking under constant and linear disturbances. The smaller SD suggests a narrower range of error fluctuations, which further confirms the excellent stability.

**Figure 15:**
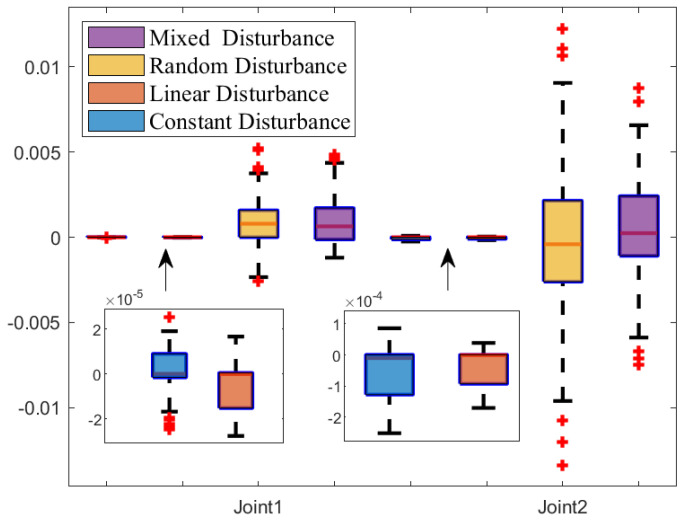
The error box plot of NRCILC-FTZNN with four different disturbances.

Figure 15 shows the tracking error box plot under different disturbances. Specifically under constant and linear disturbances, the median error is close to zero, which indicates that tracking errors are concentrated around zero. Additionally, the interquartile range under constant and linear disturbances is narrower than that of other disturbances, which suggests a lower degree of error dispersion. Therefore, constant and linear disturbances provide more stable and reliable trajectory tracking. Furthermore, shorter whisker lines under constant and linear disturbances indicate a reduction in extreme values, and fewer outliers are observed. Therefore, the distribution of tracking error data is more concentrated under constant and linear disturbances. In summary, constant and linear disturbances are characterized by tracking errors concentrated near zero, low dispersion, a concentrated data distribution, and fewer outliers.

### 5.4. System Simulation with Different Parameters

The tracking performance of the proposed scheme depends on various parameters, which makes a sensitivity analysis of these parameters essential. The NRCILC-FTZNN is evaluated for various $\beta_{1}$ and $\beta_{2}=3$, as well as various $\beta_{1}=3$ and various $\beta_{2}$ are considered. Additionally, the relevant parameters are set as γ=0.25, and $d\left(t\right)=\frac{1}{6}\pi +\frac{1}{6}\pi t+\frac{1}{6}\pi \mathrm{rand}\left(1\right)$.

**Figure 16:**
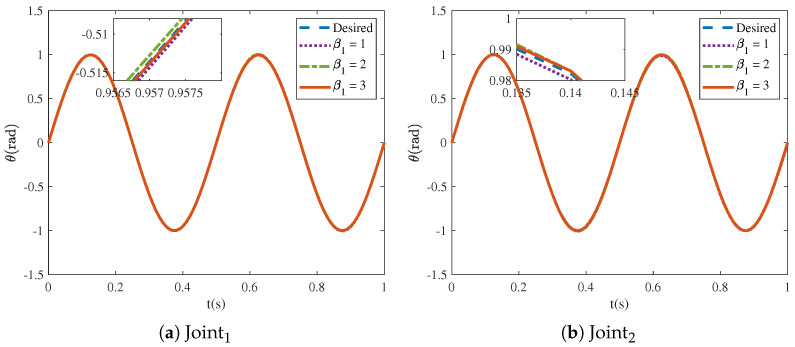
Tracking trajectories of NRCILC-FTZNN with various $\beta_{1}$.

**Figure 17:**
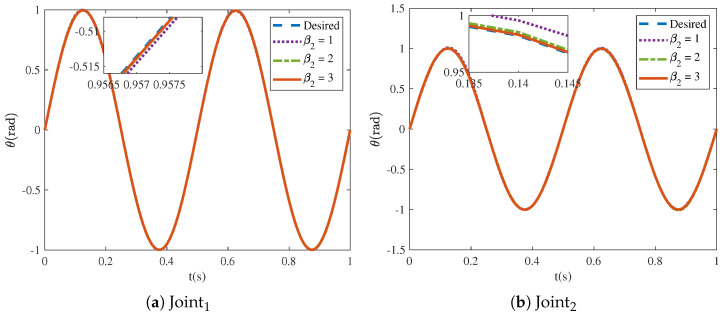
Tracking trajectories of NRCILC-FTZNN with various $\beta_{2}$.

**Figure 18:**
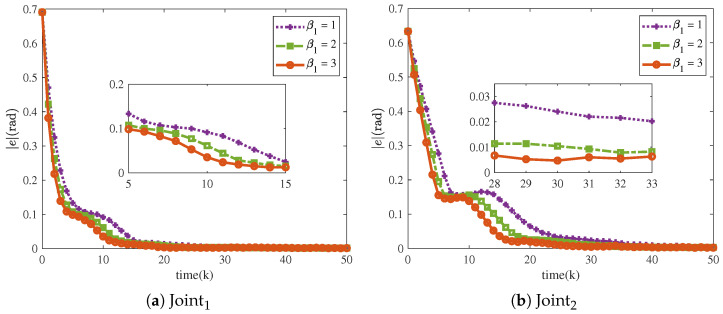
Tracking errors of NRCILC-FTZNN with various $\beta_{1}$ in iterations.

**Figure 19:**
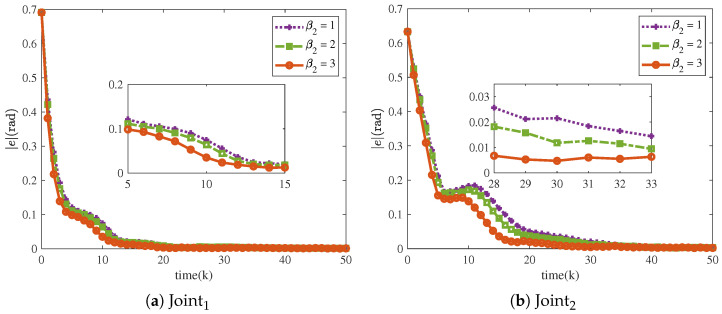
Tracking errors of NRCILC-FTZNN with various $\beta_{2}$ in iterations.

Figure 16 and Figure 17 show that the tracking trajectories of the NRCILC-FTZNN with various $\beta_{1}$ and various $\beta_{2}$ converge to the desired trajectories. Tracking errors of the NRCILC-FTZNN with various $\beta_{1}$ and various $\beta_{2}$ in iterations are shown in Figure 18 and Figure 19, respectively. As shown in Figure 18, the convergence speed of the tracking error increases with the growth of $\beta_{1}$. The weight of the linear term in the control law determines the convergence speed under large errors, and a larger $\beta_{1}$ accelerates convergence under these conditions. Furthermore, as shown in Figure 19, the convergence speed of the tracking error increases with the growth of $\beta_{2}$. The weight of the nonlinear term in the control law governs the convergence speed under small errors, and a larger $\beta_{2}$ accelerates convergence under these conditions.

The comparisons of the RMSE are shown in Table 3 and Table 4. It is clearly observed that the RMSE of ${\mathrm{Joint}}_{1}$ and ${\mathrm{Joint}}_{2}$ based on the NRCILC-FTZNN with $\beta_{1}=3$ and $\beta_{2}=3$ are 0.0017rad and 0.0031rad smaller than that under other parameter conditions. A smaller RMSE indicates that higher tracking accuracy is achieved by the NRCILC-FTZNN with larger $\beta_{1}$ and $\beta_{2}$.

**Figure 20:**
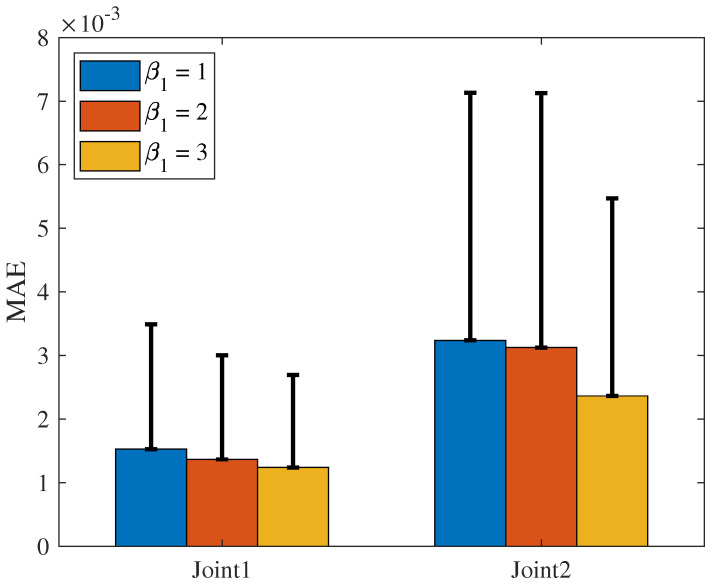
The MAE of system with various $\beta_{1}$. (The error bar is SD).

**Figure 21:**
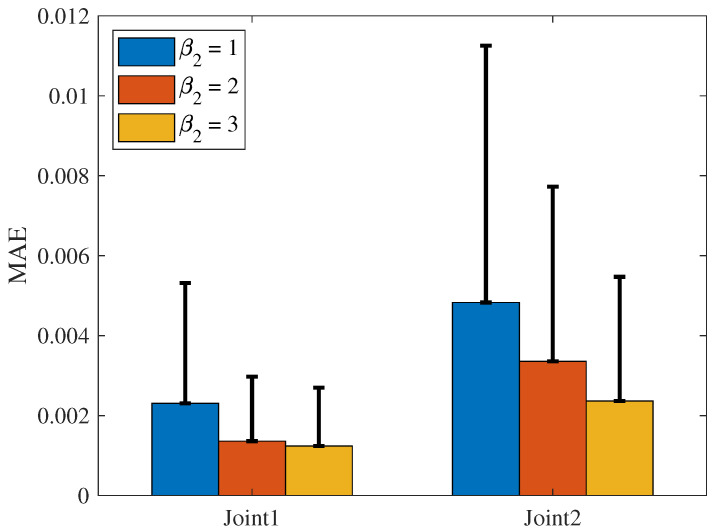
The MAE of system with various $\beta_{2}$. (The error bar is SD).

The MAE and SD of the NRCILC-FTZNN with various $\beta_{1}$ and various $\beta_{2}$ are shown in Figure 20 and Figure 21. From Figure 20, the average trajectory-tracking error of ${\mathrm{Joint}}_{1}$ and ${\mathrm{Joint}}_{2}$ with $\beta_{1}=3$, calculated by MAE, are reduced by 24.38% and 19.8% compared to the NRCILC-FTZNN with $\beta_{1}=1$ and $\beta_{1}=2$, respectively. Moreover, from Figure 21, the average trajectory-tracking error of ${\mathrm{Joint}}_{1}$ and ${\mathrm{Joint}}_{2}$ with $\beta_{2}=3$, calculated by MAE, are reduced by 49.51% and 23.56% compared to the NRCILC-FTZNN with $\beta_{2}=1$ and $\beta_{2}=2$, respectively. The MAE and SD are observed to be smaller with $\beta_{1}=3$ and $\beta_{2}=3$. The smaller MAE and SD indicate that the stability of trajectory tracking improves as $\beta_{1}$ and $\beta_{2}$ increase.

**Figure 22:**
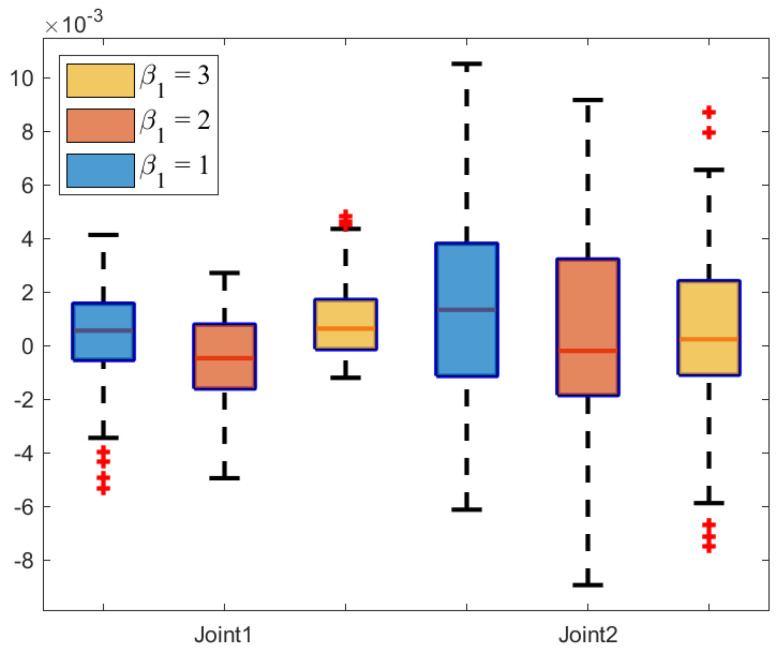
The error box plot of NRCILC-FTZNN with various $\beta_{1}$.

**Figure 23:**
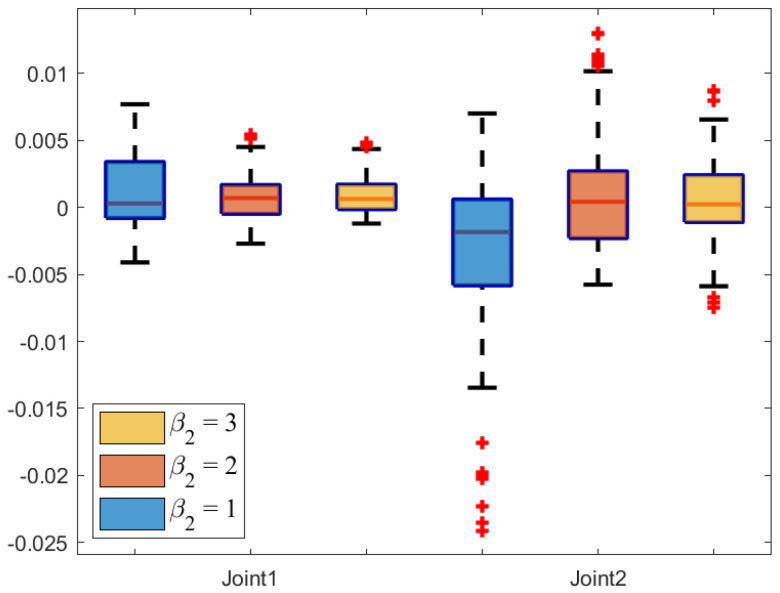
The error box plot of NRCILC-FTZNN with various $\beta_{2}$.

Figure 22 and Figure 23 illustrate the tracking error box plot for different parameter sets. When $\beta_{1}=3$ and $\beta_{2}=3$, the median error approaches zero, which indicates that the tracking errors are more concentrated near zero. Additionally, the interquartile range under $\beta_{1}=3$ and $\beta_{2}=3$ is narrower compared to other parameter conditions, which suggests a lower degree of error dispersion. Therefore, parameters set as $\beta_{1}=3$ and $\beta_{2}=3$ provide more stable and reliable trajectory tracking. Furthermore, shorter whisker lines under $\beta_{1}=3$ and $\beta_{2}=3$ indicate a reduction in extreme values. In summary, the characteristics of $\beta_{1}=3$ and $\beta_{2}=3$ include tracking errors concentrated near zero, low dispersion, and a concentrated data distribution.

### 5.5. Exploration of Experimental Implementation

To validate the practical applicability of the proposed NRCILC-FTZNN, a feasible implementation and testing plan for real hardware is outlined as follows:

(1) To explore the practical feasibility of the proposed NRCILC-FTZNN algorithm, a potential implementation plan is considered on a self-developed two-degree-of-freedom (2-DOF) planar robotic manipulator. The manipulator can be constructed using lightweight aluminum alloy to ensure both structural rigidity. Each joint is actuated by a servo motor equipped with a dedicated closed-loop driver for precise motion control. High-resolution encoders are installed at each joint to provide accurate real-time angular feedback. The control algorithm is implemented on an upper-level computer using MATLAB R2022a, which communicates with a lower-level controller, such as Arduino, and STM32, via CAN communication.
(2) The robotic manipulator is tasked with tracking a planar sinusoidal trajectory in a repetitive motion scenario. The proposed ILC without resetting conditions eliminates the need for state resetting, thereby making the control process more consistent with practical robotic operations. The control input is updated after each trial based on the tracking error using the ZNN-based learning mechanism.
(3) To simulate real-world conditions, uncertainties such as load changes and sensor noise could be introduced. For instance, small payloads may be added to the end-effector to simulate variations in system, and Gaussian noise can be injected into encoder readings to emulate measurement disturbances. These settings facilitate evaluation of the robustness and adaptability of the controller. Control performance in such a setup can be quantitatively evaluated using metrics such as RMSE, MAE, and SD of the tracking error. The indicators would include convergence speed, steady-state accuracy, and robustness under disturbances.
(4) To ensure safe execution, protective strategies such as joint limiters, control signal saturation logic, and emergency stop mechanisms should be integrated into the system. Control parameters can be conservatively tuned initially and gradually optimized once stable performance is achieved.

In summary, although physical experiments have not yet been conducted, the proposed NRCILC-FTZNN algorithm demonstrates promising potential for implementation on a self-developed 2-DOF robotic manipulator. This framework may be extended in the future to more complex platforms with higher degrees of freedom or environments involving visual feedback, compliant joints, and multi-modal sensing. Furthermore, it can be integrated with data-driven strategies such as reinforcement learning to enhance adaptability to uncertain environments.

### 5.6. Extended Controller Design

For the impact of unmodeled effects, the finite-time convergence property of the ZNN inherently enhances the ability of system to suppress transient disturbances and mitigate certain unmodeled effects. Additionally, adaptive learning mechanisms, such as iteration-based gain tuning, can be integrated into the ILC framework to accommodate nonlinear characteristics. Moreover, data-driven model estimation approaches, such as neural networks, and Gaussian processes, offer a promising means of capturing complex system that are difficult to model explicitly.

Future research will focus on these directions to extend the applicability of the NRCILC-FTZNN algorithm to more realistic robotic environments characterized by strong nonlinearities and significant uncertainties.

## 6. Conclusions

The ILC with resetting conditions based on FTZNN has been proposed for trajectory tracking of robotic manipulator under disturbances in this paper. The framework has combined a finite-time activation function of ZNN and an ILC algorithm with resetting conditions, and theoretically proved the convergence of proposed scheme. Moreover, through trajectory-tracking simulation experiments and error analysis, the superiority of the NRCILC-FTZNN in convergence has been demonstrated. Specifically, convergence analysis has shown that the NRCILC-FTZNN scheme has exhibited a rapid convergence speed. The eliminating disturbances analysis has indicated that the NRCILC-FTZNN has strong resistance to various external disturbances. Quantitative analysis has shown the superiority of the finite-time activation function of ZNN. The above error analysis has further demonstrated that the NRCILC-FTZNN has high tracking accuracy and stability.

In the future, the activation functions of the ZNN, such as the fixed-time and predetermined-time activation functions, can be replaced or optimized to address diverse applications of robotic manipulator.To further improve trajectory-tracking accuracy, advanced sensor technologies can be integrated into the control scheme. Additionally, combining the proposed approach with intelligent control algorithms, such as reinforcement learning and deep learning, can enhance the autonomous learning and adaptive capabilities of robotic manipulator. Another promising direction is applying the ILC algorithm to control robotic manipulator in real time, enabling more precise and efficient performance in practical implementations. These advancements can significantly expand the functionality and applicability of robotic manipulator systems.

## Figures and Tables

**Table 1:** RMSE of RAPD and NRCILC-FTZNN.

RAPD		NRCILC-FTZNN	
${\mathrm{Joint}}_{1}$	${\mathrm{Joint}}_{2}$	${\mathrm{Joint}}_{1}$	${\mathrm{Joint}}_{2}$
0.0176	0.0183	0.0017	0.0031

**Table 2:** RMSE Analysis of Three Schemes under Different Disturbances.

Parameters			RMSE					
$\beta_{1}$	$\beta_{2}$	Disturbance d(t)	NRCILC-LZNN		NRCILC-PZNN		NRCILC-FTZNN	
			${\mathrm{Joint}}_{1}$	${\mathrm{Joint}}_{2}$	${\mathrm{Joint}}_{1}$	${\mathrm{Joint}}_{2}$	${\mathrm{Joint}}_{1}$	${\mathrm{Joint}}_{2}$
3	3	$\frac{1}{6}\pi$	0.00035	0.00200	0.00025	0.00150	0.00001	0.00009
3	3	$\frac{1}{6}\pi t$	0.00031	0.00160	0.00022	0.00120	0.00001	0.00006
3	3	$\frac{1}{6}\pi \mathrm{rand}\left(1\right)$	0.0020	0.0051	0.0021	0.0048	0.0017	0.0047
3	3	$\frac{1}{6}\pi +\frac{1}{6}\pi t+\frac{1}{6}\pi \mathrm{rand}\left(1\right)$	0.0022	0.0065	0.0024	0.0123	0.0017	0.0031

**Table 3:** RMSE of NRCILC-FTZNN with Various $\beta_{1}$.

Parameters		RMSE	
$\beta_{1}$	$\beta_{2}$	NRCILC-FTZNN	
		${\mathrm{Joint}}_{1}$	${\mathrm{Joint}}_{2}$
1	3	0.0020	0.0043
2	3	0.0018	0.0040
3	3	0.0017	0.0031

**Table 4:** RMSE of NRCILC-FTZNN with Various $\beta_{2}$.

Parameters		RMSE	
$\beta_{1}$	$\beta_{2}$	NRCILC-FTZNN	
		${\mathrm{Joint}}_{1}$	${\mathrm{Joint}}_{2}$
3	1	0.0032	0.0073
3	2	0.0018	0.0045
3	3	0.0017	0.0031

## Data Availability

The original contributions presented in this study are included in the article. Further inquiries can be directed to the corresponding author.
